# Rural Water Supplies

**Published:** 1907-01-19

**Authors:** 


### RURAL WATER SUPPLIES.

ON November 28 the members of the Rural Housing and Sanitation Association met in the Caxton Hall, Westminster, to listen to and discuss an address by Dr. John Thresh, Lecturer on Public Health at the London Hospital, on rural water supplies. The chair was taken by the Earl of Dundonald, who deplored the increasing depopulation of the country districts, and even suggested that outside pressure might be employed to correct the growing disproportion between town and country population.

Dr. Thresh founded his remarks on the results of the Public Health Acts of 1875 and 1878, which empowered the sanitary authorities to provide water supplies for their districts. In urban areas this power has been successfully used, but the same cannot be said for country districts. The question is largely one of expense. The first essential is that the authorities should have an accurate knowledge of the condition of the existing water-supply, and though they have the power to expend money in investigating this, they very rarely do so. Where it cannot be absolutely shown that cases of disease are due to some fault or deficiency in the water-supply, it is assumed that all is satisfactory. There is, indeed, a popular notion that water from shallow wells is polluted, and may cause typhoid fever, and this keeps many people from living in the country. But in Dr. Thresh's opinion there is no need for this fear, if only the wells are properly constructed, but unfortunately this is not by any means universal. Moreover, even if typhoid does not result from pollution of the water-supply, it does not follow that no injury to health is to be feared, and in places where dairy farming is carried on, or where it is desired to establish factories, or in any other way find work for the people, a good water supply is essential.

At present the owner of a house is not compelled to provide a water supply for it if the cost of doing so exceeds £8 13s. 4d., though with the consent of the Local Government Board he may expend as much as £13. Clearly this limitation makes it difficult for the authorities to bring sufficient pressure to bear on property owners to compel them to provide the requisite water supply. Nor have the authorities the power to make an owner or owners provide a single common source of supply for a group of houses which are reasonably near each other, and apportion the cost among them, unless the water can be laid to each house, which would often mean a very serious expense. Even if existing houses were left unprovided with a convenient supply of good water, it might be supposed that when new ones are built this important matter would be seen to. In intention it is. There is a penalty of £10 demanded for occupying a new house without obtaining a certificate that it is supplied with water, but as this is not a recurring penalty, many builders find it cheaper to pay it once for all than to provide the water. When this penalty has once been paid the only steps which the authorities can take are those allowed for dealing with occupied houses, *i.e.* the £8 to £13 limit.

It is true that the Local Government Board can compel the rural sanitary authorities to provide a water supply, but it must first be shown that the existing conditions threaten a danger to the health of the inhabitants, and that a proper supply can be got at at a "reasonable cost"—a very vague definition. Moreover the rural authorities have good reason to fear going to the Local Government Board for permission to improve their water supply. That body does not always seem to realise that the perfection of works which is needed in a town is by no means always necessary in a country district. Thus in town it may be necessary to provide all machinery in duplicate, for the consequences of an interruption of the water supply, affecting a large number of people might be very serious. But in a sparsely populated district they would not be equally serious, and it is better to have a scheme which might on a rare occasion be unsatisfactory than to have no scheme at all. Moreover, the rural authorities have learned to distrust the Local Government Board. Dr. Thresh says: "The opinion is universal, though I do not assert that it is now well-founded, that the more expensive a scheme the more likely it is to receive the sanction of the Board, and that however expensive it is, the Board will require additions and alterations which will greatly add to that expense."

Dr. Thresh thinks it would be wiser for authorities to consider comprehensive schemes for supplying a number of parishes rather than a series of small local supplies. The former course would be more economical, and would be more easily supervised. This would imply power being vested in some authority of wider scope than the parish council, of whom, from the point of view of a sanitarian, Dr. Thresh does not think highly. Parish councillors, often themselves owners of property, are not keen to take up the ungracious task of forcing property owners to provide a water supply, and it is unfortunate that County Councils have no power to take action unless such action is demanded by a resolution of the parish council. If the County Council had the power to take the initiative even in pressing the parish authorities to protect wells, streams, and springs from pollution, and keep the parish pumps in proper repair, it would be a good thing.

Another point was raised by Mr. A. H. Matthews, Secretary of the Central Chamber of Agriculture, who touched on the unsatisfactory position of medical officers of health who, appointed by the local authorities, have not the power they need to insist on improvements in their districts. Mr. Matthews' suggestion that the public health service should form a branch of the Civil Service would give all sanitary officers, from the M.O.H. downwards, far more independence.

Miss Constance Cochrane, who is so well known in connection with rural housing and its defects, pointed out another difficulty. In London, and also in Scotland, "any individual" has the power to complain of bad conditions which have not received proper attention at the hands of owners and sanitary authorities, while in the English counties the complaint must come either from the sufferer or from "two inhabitant householders." The aggrieved person knows that if he makes a complaint the chances are that he will be told to quit, and he may not find it easy to get another house, and the "inhabitant householders" are not likely to set the law in operation against fellow-owners of property. Among other things Miss Cochrane spoke of the importance of providing large gardens—not less than a quarter of an acre—for every cottage, so that where there are no drains refuse of all kinds can be disposed of without injury to health. The meeting proved how many difficulties stand in the way of the satisfactory housing of the poor in country districts, but also showed that the Association had a practical knowledge of them and practical suggestions to offer as to their removal.

